# Natural OX40L expressed on human T cell leukemia virus type-I-immortalized T cell lines interferes with infection of activated peripheral blood mononuclear cells by CCR5-utilizing human immunodeficiency virus

**DOI:** 10.1186/1743-422X-10-338

**Published:** 2013-11-18

**Authors:** Daigo Kasahara, Azusa Takara, Yoshiaki Takahashi, Akira Kodama, Reiko Tanaka, Aftab A Ansari, Yuetsu Tanaka

**Affiliations:** 1 Department of Immunology, Graduate School of Medicine, University of the Ryukyus, Okinawa 903-0215, Japan; 2 Department of Pathology, Emory University School of Medicine, Atlanta, GA 30322, USA

## Abstract

**Background:**

OX40 ligand (OX40L) co-stimulates and differentiates T cells via ligation of OX40 that is transiently induced on T cells upon activation, resulting in prolonged T cell survival and enhanced cytokine production by T cells. This view has led to the targeting of OX40 as a strategy to boost antigen specific T cells in the context of vaccination. In addition, the ligation of OX40 has also been shown to inhibit infection by CCR5-utilizing (R5) but not CXCR4-utilizing (X4) human immunodeficiency virus type-1 (HIV-1) via enhancement of production of CCR5-binding β-chemokines. It was reasoned that human T cell leukemia virus type-I (HTLV-1) immortalized T cell lines that express high levels of OX40L could serve as an unique source of physiologically functional OX40L. The fact that HTLV-1^+^ T cell lines simultaneously also express high levels of OX40 suggested a potential limitation.

**Results:**

Results of our studies showed that HTLV-1^+^ T cell lines bound exogenous OX40 but not OX40L, indicating that HTLV-1^+^ T cell lines express an active form of OX40L but an inactive form of OX40. Anti-OX40 non-blocking monoclonal antibody (mAb), but not blocking mAb, stained HTLV-1^+^ T cell lines, suggesting that the OX40 might be saturated with endogenous OX40L. Functionality of the OX40L was confirmed by the fact that a paraformaldehyde (PFA)-fixed HTLV-1^+^ T cell lines inhibited the infection of autologous activated peripheral blood mononuclear cells (PBMCs) with R5 HIV-1 which was reversed by either anti-OX40L blocking mAb or a mixture of neutralizing mAbs against CCR5-binding β-chemokines.

**Conclusions:**

Altogether, these results demonstrated that autologous T cell lines immortalized by HTLV-1 can be utilized as a conventional source of physiologically functional OX40L.

## Background

OX40 ligand (OX40L, CD252) belonging to the tumor necrosis factor (TNF) superfamily is a co-stimulatory molecule [1,2] that was first described by our laboratory as gp34 that is constitutively expressed at high levels on the surface of human T cell leukemia virus type-I (HTLV-1)-immortalized T cell lines [3,4]. It is now clear that OX40L can be induced on a wide variety of human hematopoietic cell lineages including antigen presenting cells (APCs) such as dendritic cells (DCs) [5] and B cells [6], natural killer (NK) cells [7], mast cells [8], endothelial cells [9] and T cells [10,11]. OX40 (CD134), a member of the TNF receptor (TNFR) superfamily that is rapidly induced predominantly on T cells upon cell activation is the cognate receptor for OX40L [12-14]. Interaction of OX40 on T cells with OX40L on APCs generates a variety of biological changes that include enhanced production of cytokines by T cells, Th2 cell differentiation, prolonged T cell survival, activation of B cells and DCs, to name a few [1,12,15]. OX40L is naturally expressed on the cell surface as a trimeric protein that binds to three copies of monomeric OX40 within close proximity [16]. Such close interactions between OX40/OX40L promotes tight cell to cell adhesion facilitating T cell-DC communication and skin infiltration of OX40^+^ leukemic T cells in adult T cell leukemia (ATL) [17].

It has been proposed that the targeting of OX40 on activated T cells by OX40L or with the use of anti-OX40 agonistic antibodies may provide a strategy for the selective expansion of the limited frequencies of antigen specific T cells that are normally induced during vaccination and thereby achieve more effective immune responses [18-20]. Another immunological role of OX40L-OX40 interaction that we have previously documented includes the ability of OX40L in either soluble or membrane-bound form to effectively inhibit the infection of activated PBMCs with R5 HIV-1 *in vitro*[21]. This inhibition was shown to be mediated via the enhanced production of the CCR5-binding β-chemokines that include RANTES, MIP-1α and MIP-1β, followed by the down-modulation of cell surface CCR5 expression. These findings brought into focus the potential use of OX40L as a therapeutic tool and prompted us to investigate methodologies that would provide a convenient source for biologically active OX40L. One such source of OX40L was reasoned to be HTLV-1^+^ T cell lines that unlike normal activated T cells or non-T cells have been shown to express both OX40L and OX40 on the cell surface at a single cell level due to the action of the HTLV-1-encoded oncogenic protein Tax [4,22]. Tax, in addition, also induces the expression of 4-1BB and its cognate ligand both of which belong to the TNF/TNFR family [23]. Selective induction of these ligand/receptor pairs has been implicated in the survival of HTLV-1-infected cells.

Studies were therefore carried out in efforts to examine whether OX40L and OX40 were expressed in a biologically active form by HTLV-1^+^ T cell lines. We report herein for the first time that HTLV-1^+^ T cell lines express a biologically active form of OX40L while the OX40 molecule appears biologically inactive or masked. The OX40L expressed by HTLV-1^+^ T cell lines was capable of inhibiting R5 HIV-1 infection of activated PBMCs via production of CCR5-binding β-chemokines. These findings suggest that autologous HTLV-1-immortalized T cell lines can be utilized as a readily available convenient source of natural OX40L in large quantities for various immunological studies.

## Results

### HTLV-1-immortalized T cell lines express active OX40L together with inactive OX40

**Figure 1:**
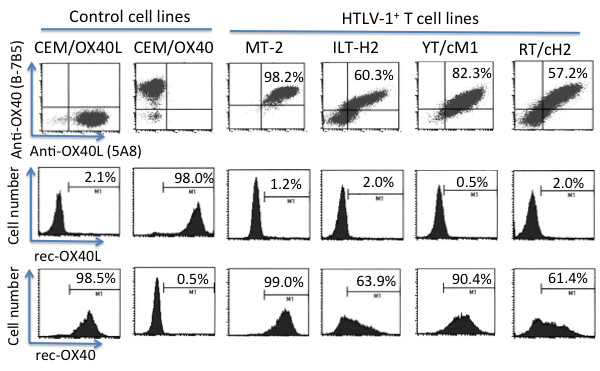
**HTLV-1**^**+**^**T cells co-express both OX40L and OX40 but only OX40L is expressed in an active form.** The OX40 and OX40L co-expressing control CEM cells and the HTLV-1^+^ cells were dually stained with FITC-labeled anti-OX40 (B-7B5) and Cy-5 labeled anti-OX40L (5A8) (upper row), or singly stained either with biotinylated recombinant OX40L (rec-OX40L) or rec-OX40 followed by PE-streptavidin (middle and lower rows, respectively). Data shown are representative profiles of 3 independent experiments.

In order to determine whether OX40L and OX40 co-expressed on the cell surface of HTLV-1^+^ T cell lines were biologically active, we examined their capacities to bind biotinylated rec-OX40 and rec-OX40L, respectively. The finding that rec-OX40 and rec-OX40L bound specifically to the OX40L-transfected CEM cells (CEM/OX40L) and the OX40-transfected CEM/OX40 cells, respectively, demonstrated the specificity of the assay being utilized (Figure 1). Interestingly, although the standard HTLV-1^+^ T cell line (MT-2) was stained double positive with anti-OX40L (clone 5A8) and anti-OX40 (clone B-7B5) mAbs, they bound only rec-OX40 but not rec-OX40L. This finding indicated that while OX40L was expressed in an active form on MT-2 cells, the OX40 was likely to be expressed in an inactive form. Similar results were obtained by the testing of a number of additional HTLV-1^+^ T cell lines, including T cell lines spontaneously established from a HTLV-1-infected patient with adult T cell leukemia (ILT-H2) and a HTLV-1-associated myelopathy (HAM/TSP) patient (ILT-M1), and various *in vitro*-HTLV-1-immortalized CD4^+^ or CD8^+^ T cell lines from different healthy donors (such as YT/cM1, RT/cH2 cells) (Figure 1). Thus, these results suggest that on the cell surface of the HTLV-1^+^ T cell lines only OX40L, but not OX40, is capable of binding its respective ligand.

### Characterization of OX40 on HTLV-1^+^ T cells

**Figure 2:**
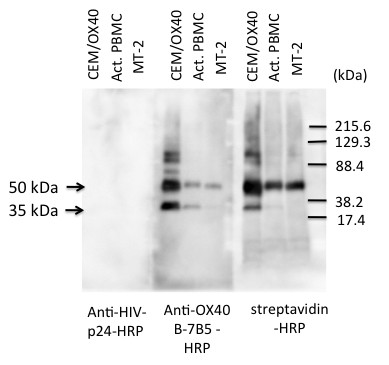
**Western blot analysis of OX40.** OX40-expressing CEM cells (CEM/OX40), *in vitro* activated PBMCs and MT-2 cells were cell-surface labeled with biotin, lysed and immunoprecipitated with anti-OX40 (B-7B5). The precipitates were subjected to 10% PAGE and blotted onto nitrocellulose sheets. The sheets were then probed with HRP-labeled anti-HIV-1 p24 (as a control), anti-OX40 (B-7B5) or streptavidin. Mol. Wt. markers are shown on the right. Data shown are representative of 3 independent experiments.

A series of studies were subsequently conducted in efforts to identify the potential reason(s) for the failure of HTLV-1^+^ T cell lines to bind rec-OX40L. Western Blot analysis of OX40 expressed by HTLV-1^+^ T cell line was first carried out to determine whether the OX40 expressed by these cells was truncated. Cell lysates prepared from surface biotinylated *in vitro* activated PBMCs and the OX40 transfected CEM cell line (CEM/OX40) were analyzed in parallel with the HTLV-1^+^ T cell line MT-2 using standard Western Blot techniques. Results of these studies displayed in Figure 2 showed that there were no detectable differences in the molecular weight of the glycosylated authentic OX40 (50 kDa) among these three samples. The 35 kDa band corresponding to the non-glycosylated form of OX40 was apparent in CEM/OX40 cells and activated PBMCs, but it was faint in MT-2 cells. These data indicated that there was no detectable deletion or modification in the glycosylated OX40 molecules expressed by the HTLV-1^+^ T cell lines.

**Figure 3:**
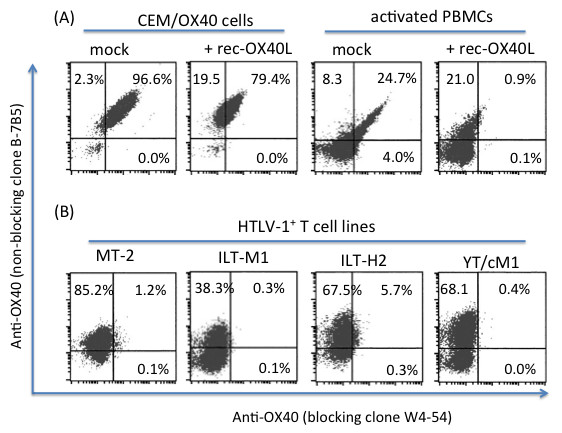
**Blocking (clone W4-54) versus non-blocking (clone B-7B5) mAb against 2 distinct epitopes of OX40 distinguish between OX40L bound and unbound OX40. (A)** OX40-expressing CEM and activated PBMCs were stained with the two mAbs in the absence (mock) or presence of 1 μg/ml of recombinant OX40L (rec-OX40L). **(B)** Various HTLV-1^+^ T cell lines were stained with B-7B5 andW4-54 labeled with FITC and Cy5, respectively. Data shown are representative of 3 independent experiments.

**Figure 4:**
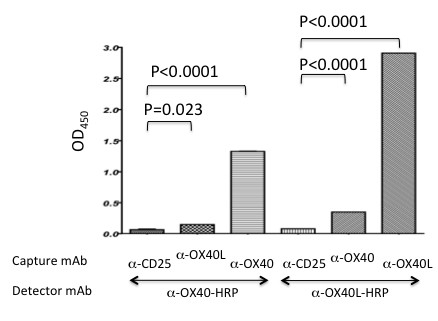
**Presence of OX40-OX40L complexes in HTLV-1**^**+**^**T cell lysates.** The ILT-H2 cell line derived from an ATL patient were lysed and the lysates incubated in microtiter wells that had been previously coated with either anti-CD25, OX40 or OX40L mAb for 1 hour. Anti-CD25 mAb was used as a non-specific negative control. After washing, the levels of OX40 or OX40L bound to the plates were assayed using either HRP-labeled anti-OX40 or anti-OX40L mAb. Data shown are representative of 3 independent experiments.

To further probe for the molecular basis for the inability of the OX40 expressed by the HTLV-1^+^ T cell lines to bind rec-OX40L, we utilized an additional anti-OX40 specific mAb (W4-54 mAb) along with B-7B5 mAb. While the clone W4-54 anti-OX40 mAb inhibited the binding of OX40 and OX40L, the clone B-7B5 failed to show any detectable inhibition (Additional file 1: Figure S1). These two mAbs are reasoned to react against conformational epitopes since they failed to bind any overlapping 15-mer peptides spanning the entire OX40 protein (data not shown). As shown in Figure 3(A), control mock treated CEM/OX40 and activated PBMCs, as expected, both stained dual-positive with the B-7B5 mAb and W4-54 mAbs. These data show that the comparative staining with B-7B5 and W4-54 mAbs can be potentially utilized to distinguish between non-ligated versus OX40L ligated forms of OX40. Figure 3(B) shows that although B-7B5 mAb stained HTLV-1^+^ T cell lines at high levels, little or no staining was noted with the use of the W4-54 mAb. In contrast, results of a WB analysis showed that the W4-54 mAb readily reacts to the p50 of the OX40 molecule in lysates of the HTLV-1^+^ T cell line, YT/cM1 (Additional file 2: Figure S2). These results suggest that the OX40L binding site of OX40 expressed by the HTLV-1^+^ T cell lines was altered, most probably due to pre-occupation with endogenous OX40L. To confirm this possibility, we explored the presence of OX40-OX40L complexes expressed by HTLV-1^+^ T cell lines using our in-house ELISA. Cell lysates of the ATL-derived HTLV-1^+^ T cell line (ILT-H2) were first captured with the use of immobilized anti-OX40L (clone HD-1) or anti-OX40 (clone B-7B5) mAb, respectively. The levels of captured antigens were assayed with the use of HRP-labeled anti-OX40 mAb or anti-OX40L mAb. Although it is reasonable to assume that the natural interaction between OX40 and OX40L on the living cell surface may be dissociated by the detergent treatment, as shown in Figure 4, low but significant levels of OX40-OX40L complex were still detectable in the cell lysates.

### Functional OX40L expressed by HTLV-1^+^ T cell lines

**Figure 5:**
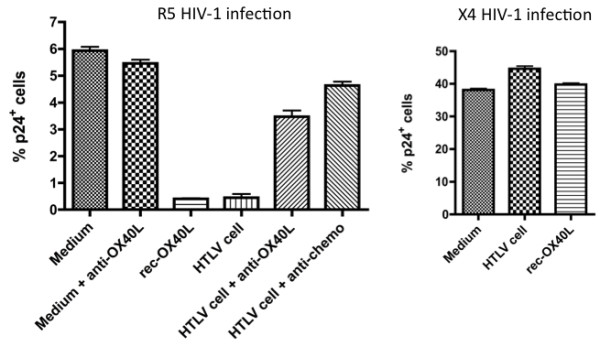
**PFA-inactivated HTLV-1**^**+**^**T cells inhibit infection of activated autologous PBMCs with R5 HIV-1, but not X4 HIV-1, via OX40L and β-chemokines.** In vitro activated PBMCs were infected with either R5 HIV-1 (JR-FL strain) or X4 HIV-1 (NL4-3 strain) and cultured in the presence or absence of recombinant OX40L, PFA-inactivated autologous HTLV-1^+^ T cells, anti-OX40L blocking mAb (5A8) or a mixture of anti-β-chemokine neutralizing mAbs. After 4 days, the cells were examined for intracellular HIV-1 p24 by FCM. Data shown are representative of 3 independent experiments.

To confirm that the OX40L expressed on the HTLV-1^+^ T cell lines is biologically functional, we performed co-culture experiments using the experimental *in vitro* infection of autologous activated PBMCs with HIV-1 as a read out. PBMCs activated with anti-CD3/anti-CD28 mAbs for 24 hours were washed and infected with either R5 HIV-1_JR-FL_ or X4 HIV-1_NL4-3_ at a low m.o.i., and then co-cultured with paraformaldehyde (PFA)-fixed autologous HTLV-1^+^ T cell line in the presence or absence of anti-OX40L mAb or a mixture of the three CCR5-binding chemokine-blocking mAbs (anti-RANTES, anti-MIP-1α and anti-MIP-1β). The reasons why we utilized autologous PFA-fixed HTLV-1^+^ T cell lines were to avoid any allogeneic stimuli and minimize the secretion of any anti-HIV-1 factors by the HTLV-1^+^ T cell lines. As shown in Figure 5, the frequencies of HIV-1 p24^+^ T cells in the cultures were reduced by co-culture with not only autologous HTLV-1^+^ T cell line but also with the addition of soluble rec-OX40L. This inhibition was mediated by OX40L-OX40 interaction since the addition of the anti-OX40L blocking mAb (clone 5A8) and/or the addition of a mixture of the anti-β-chemokine mAbs reversed the level of reduction. It is worthy to note that, similar to data we have previously reported with the use of recombinant OX40L [21], X4 HIV-1 infection was not influenced by co-cultivation with PFA-fixed HTLV-1^+^ T cell line, suggesting the CCR5-specificity of this antiviral effect.

**Figure 6:**
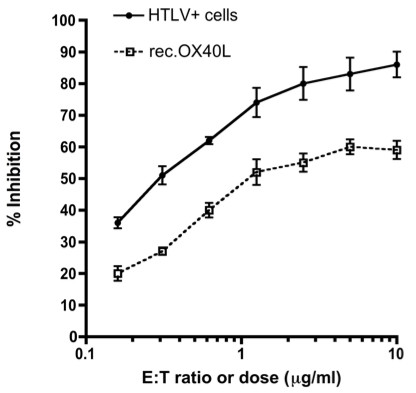
**HTLV-1**^**+**^**T cells are more potent in the inhibition of R5 HIV-1 infection than recombinant soluble OX40L.** R5 HIV-1-infected PBMCs prepared as in Figure 5 were cultured in the presence or absence of a graded concentration of recombinant soluble OX40L or PFA-fixed autologous HTLV-1^+^ T cells. After 4 days, the levels of p24 produced in the culture supernatants were quantitated by ELISA. Data shown are representative of 2 independent experiments.

Finally, we compared the potential of membrane bound OX40L of the fixed HTLV-1^+^ T cell lines with that of soluble rec-OX40L to inhibit R5 HIV-1 infection by the quantitation of p24 production in the culture supernatants. As shown in Figure 6, whereas the inhibitory effect of the rec-OX40L reached a plateau at levels > 1.25 μg/ml, the autologous HTLV-1^+^ T cell line could inhibit more effectively at even an HTLV-1^+^ T cell to PBMCs ratio as low as 0.3. The maximum inhibition reached with rec-OX40 was around 65% of the maximum inhibition reached with HTLV-1^+^ T cell line, with similar IC_50_. Altogether, these data demonstrate that indeed, the OX40L expressed by HTLV-1^+^ T cell lines is biologically active.

## Discussion

In the present study, we revealed that the cell surface expressed OX40L on T cell lines immortalized by HTLV-1 is biologically active in concert with the co-expression of an inactive form of OX40. As far as we know, this is the first study to report the polarized “OX40L-active/OX40 inactive” expression by HTLV-1^+^ T cell lines. The expression of active forms of OX40L is not unique to HTLV-1^+^ T cell lines, since similar conditions have also been observed in normal T cells when they are activated under mild DNA damaging conditions or cultured for long-term in IL-2 containing media with periodic stimulation [11,24]. However, compared to these normal T cells, HTLV-1^+^ T cell lines are immortal and thus can provide unlimited amounts of OX40L.

The precise mechanism for the inability of OX40 on HTLV-1^+^ T cell lines to bind OX40L remains to be clearly defined. Based on our previous paper showing that functional OX40L can be transferred to OX40 intercellularly [25], we hypothesize that the cell surface OX40 may be saturated with endogenously produced OX40L in *cis* and/or *trans* mode. Indeed, the WB analysis showed that the OX40-OX40L blocking mAb W4-54 that did not stain living HTLV-1^+^ T cells reacted to the p50 OX40 molecule (Additional file 2: Figures S2 and Additional file 3: Figures S3). In accordance with this assumption, we demonstrated the presence of OX40-OX40L complexes in lysates of HTLV-1^+^ T cell lines by ELISA (Figure 4). It remains unclear why there were significant amounts of OX40L-free OX40 molecules in the lysates from HTLV-1^+^ T cells as determined by ELISA. It will be highly likely that the detergent treatment dissociates the OX40 and OX40L complex due to perturbation of cytoplasmic membrane structure including lipid rafts on which OX40 is supposed to reside in association with the other TNFR member such as 4-1BB [26,27]. In addition, our preliminary data that supports the OX40 saturation hypothesis includes the finding using the HUT 102 cell line that is another HTLV-1^+^ T cell line from which the original OX40 gene was cloned [13]. This HUT102 cell line stained with both B-7B5 and W4-54 mAbs, but not with anti-OX40L (5A8 mAb), and was able to bind recombinant OX40L but not OX40 (Additional file 3: Figure S3). Although it is not clear why HUT102 cell line was positive for Tax antigen but negative for OX40L expression, these data clearly showed that in the absence of OX40L, functional OX40 can be expressed on the cell surface. It will be of interest to examine whether the inactive form of the OX40 can be converted to an active form after silencing the expression of OX40L in HTLV-1^+^ T cell lines. Such studies are currently in progress.

On the basis of the present and previous results on OX40L [21], it can be hypothesized that OX40L may have a therapeutic and prophylactic potential against R5 HIV-1 infection. However, at present, purified biologically active forms of human OX40L protein in large quantities is not available. The alternative is to utilize OX40L-fusion proteins [28], OX40L-expressing recombinant virus [20], OX40L mRNA-transfected cells [29], lentivirus-transduced DCs [30], or autologous dying normal T cells [24]. The superiority of using cell membrane-bound OX40L as compared with the use of a soluble form was documented by data observed by the degree of inhibition of R5 HIV-1 as seen in the present study (Figure 6). These findings are in accord with a previous study that showed that the membrane-immobilized form of OX40L is highly active in the stimulation of an OX40-transfected cell line to produce cytokines [31]. In addition to OX40L, HTLV-1^+^ cell lines may exert additional suppressing effect on R5 HIV-1 infection via Tax protein, since Tax proteins of HTLV-1 and HTLV-2 have been shown to play a role in generating antiviral responses against HIV-1 via induction of CCR5-binding chemokines *in vitro*[32]. This view is supported by the finding that co-infection with HTLV interferes with the progression of HIV-1 disease *in vivo*[33]. However, such Tax effects in the present study may be less potent than OX40L since anti-OX40L mAb significantly reversed the suppression of R5-HIV-1 induced by co-culture with autologous HTLV-1^+^ T cell lines (Figure 5).

## Conclusions

The present results demonstrate that HTLV-1^+^ T cell line is a unique source of functional human OX40L, and suggest that autologous HTLV-1-immortalized T cell lines can be utilized as a conventional source of natural and functional OX40L in large quantities for various immunological studies.

## Methods

### Reagents

The medium used throughout the studies consisted of RPMI 1640 medium (Sigma-Aldrich. Inc. St. Louis, MO), supplemented with 10% fetal calf serum (FCS), 100 U/ml penicillin and 100 μg/ml streptomycin (hereinafter called RPMI medium). Anti-human CD3 mAb (clone OKT-3) and agonistic anti-CD28 mAb were purchased from the American Type Culture Collection (Rockville, MD) and Biolegend (San Diego, CA), respectively. Neutralizing mAbs against human RANTES, MIP-1α, and MIP-1β were purchased from R&D systems (Minneapolis, MN). The mouse mAbs produced in our laboratory included anti-OX40L (blocking clone 5A8 [34] and clone HD1, unpublished), anti-human OX40 (non-blocking clone B-7B5 and clone 17D8 [35]), anti-HIV-1 p24 (clones NP-24 and 2C2 [21]) and anti-CD25 (clone H-8) [36]. The rat mAbs included anti-human OX40 (blocking clone W4-54) and anti-HCV (clone Mo-8) [25,37]). Some clones were labeled with HRP using a kit (Dojin, Kumamoto, Japan) and used as the detector mAb in ELISA. These in-house mAbs were isolated from ascites fluid prepared in Balb/c or CB.17-SCID mice. The IgGs were purified utilizing a standard gel filtration method. Some of them were labeled with FITC, HiLyte Fluor 647 or Cy5 using commercial labeling kits (Dojin, GE Healthcare) according to the manufacturer’s instructions. Biotinylated recombinant-soluble human OX40 (sOX40 in a form of murine IgG2a-Fc fusion protein) and OX40L (sOX40L in a form of murine CD8-fusion protein) were purchased from Ancell (Bayport, MN) and used with PE-streptavidin (BioLegend) for staining. Unlabeled glycosylated recombinant human OX40L that consists of OX40L with a human CD33 signal peptide produced in NS1 cells was purchased from R&D systems. Human recombinant IL-2 was obtained as a courtesy from the NIH-AIDS Reagent and Repository program (Bethesda, MD).

### Cell lines

The HTLV-1-producing T cell lines used included the MT-2, HUT102 and the IL-2 dependent T cell lines ILT-M1 and ILT-H2 that had been generated from a HTLV-1-associated myelopathy (HAM) and an adult T cell leukemia (ATL) patient, respectively. Additional cell lines utilized included the CEM cell lines transfected with either human OX40L or OX40 (CEM/OX40L and CEM/OX40) [38]. T cells isolated from normal human donors were immortalized by HTLV-1 as follows. PBMCs from healthy donors were obtained by density gradient centrifugation of heparinized whole blood on HistoPAQUE-1077 (Sigma-Aldrich), suspended at 2 × 10^6^ cells/ml in RPMI medium, dispensed into individual wells of 24-well plates (BD) (1 ml/well) pre-coated with 5 μg/ml OKT3 for 1 hour and cultured in the presence of soluble 0.1 μg/ml anti-CD28 mAb. After 24 hours at 37°C in a 5% CO_2_ humidified atmosphere, the activated PBMCs were harvested and washed once. These activated PBMCs (1 × 10^6^ cells/ml) were mixed with an equal number of ILT-M1 cells that were pretreated with 50 μg/ml MMC for 30 min at 37°C and cultured in RPMI media supplemented with 20 U/ml IL-2 (culture media). The cultures were performed in 24-well plates (BD) (2 ml/well) and the culture media was replenished every 3–4 days. After 1 ~ 2 months when HTLV-1 Tax^+^ T cells appeared and started to grow continuously, they were split every 3 to 5 days using the culture medium.

### Flow Cytometry (FCM)

FCM analysis of live cells was carried out as described previously. Briefly, cells to be analyzed were Fc-blocked with 2 mg/ml normal human pooled IgG on ice for 15 min. Aliquots of these cells were then subjected to staining using pre-determined optimum concentrations of fluorescent dye-conjugated mAbs for 30 min on ice. The cells were then washed using FACS buffer (PBS containing 2% FCS and 0.1% sodium azide), fixed in 1% paraformaldehyde (PFA) containing FACS buffer and analyzed using a FACS Calibur, and the data obtained were analyzed using the Cell Quest software (BD). In order to determine whether cell surface OX40 or OX40L is functional, aliquots of Fc-blocked cells were incubated with either biotinylated recombinant-OX40L (rec-OX40L) or rec-OX40 at a concentration of 2.5 μg/ml for 30 minutes on ice, followed by staining with PE-labeled streptavidin (Beckman Coulter) for 30 minutes on ice and then analyzed by FCM. For detection of HIV-1 infected cells, PBMCs were fixed with PBS containing 4% PFA followed by washing twice in FACS buffer containing 0.5% saponin. These cells were Fc-blocked with 2 mg/ml normal human pooled IgG on ice for 15 min, and aliquots of these cells were stained with FITC- or Cy5-conjugated anti-HIV-1 p24 mAb (clone 2C2) for 30 min on ice. The cells were then washed using FACS buffer and absolute cell counts of p24^+^ cells were performed by FCM using a cell counting kit (BD) according to the manufacturer’s protocol. For staining of Tax antigen, cells were fixed with PBS containing 4% PFA followed by washing in FACS buffer containing 0.5% saponin. Aliquots of these cells were stained with Cy5-conjugated mouse anti-Tax mAb (Lt-4) [39] for 30 min on ice.

### ELISA and Western blot

For the quantitation of OX40L and OX40 by ELISA, anti-OX40L capture mAb (clone HD1)/ HRP-labeled detector mAb (clone 8F4) and anti-OX40 (clone B-7B5)/HRP-labeled detector mAb (clone 17D8), respectively, were used together with recombinant standard proteins purchased from R&D systems. Immunoprecipitation followed by Western blot analysis of OX40 was performed as reported previously [40].

### HIV-1 preparation and infection

HIV-1_JR-FL_ and HIV-1_NL4-3_ viral stocks were produced as described previously [21]. *In vitro* activated PBMCs were prepared as described above, washed once and infected with either R5 HIV-1_JR-FL_ or X4 HIV-1_NL4-3_ at a multiplicity of infection (m.o.i.) of 0.005 for 2 hours. After washing 3 times, PBMCs were re-suspended at 1 × 10^6^ cells/ml in 20 U/ml IL-2-containing RPMI medium, dispensed into individual wells of 48-well plates (BD) (0.5 ml/well) and cultured in the presence or absence of 1 μg/ml of rec-OX40L or graded numbers of autologous HTLV-1^+^ T cells (HTLV-1^+^ T cells : PBMCs ratio of 10 to 0.15) that had been previously inactivated with 4% paraformaldehyde (PFA). Production of HIV-1 was determined by either the measurement of HIV-1 core p24 levels produced in the culture supernatants using our in-house formulated and standardized kits or FCM using Cy5 labeled anti-HIV-1 p24 mAb [21].

### Statistical analysis

Data were tested for significance using the Student’s *t* test by using Prism software (GraphPad Software).

## Competing interests

The authors declare no competing financial interests.

## Authors’ contributions

DK and YTak generated HTLV-1^+^ T cell lines and carried out the FCM and ELISA, performed the statistical analysis and drafted the manuscript. AT performed WB and FCM analyses. AK produced R5 and X4 HIV-1 and titrated. RT produced and labeled antibodies, confirmed their specificities and made in-house ELISA. AAA participated in the design of the study and helped to draft the manuscript. YT conceived of the study, participated in its design and coordination, carried out the HIV-1 infection experiments and drafted the manuscript. All authors read and approved the final manuscript.

## Supplementary Material

Additional file 1: Figure S1 Characterization of two anti-human OX40 mAbs. In the presence of B-7B5, W4-54 or isotype control mAbs, the OX40 and OX40L co-expressing control CEM cells were singly stained either with biotinylated recombinant OX40L (rec-OX40L) or rec-OX40, respectively, followed by PE-streptavidin. Data shown are representative profiles of 3 independent experiments. Click here for file

Additional file 2: Figure S2 Detection of OX40 expressed by HTLV-1^+^ T cell line (YT/cM1) by Western Blot with B-7B5 and W4-54 mAbs. Cell lysates of HTLV-1^+^ T cell line, YT/cM1, were subjected to 10% PAGE and blotted onto nitrocellulose sheets. The sheets were then probed with anti-OX40 mAbs (B-7B5 or W4-54) or isotype controls followed by goat anti-mouse IgG or anti-rat IgG. Mol. Wt. markers are shown on the right. Data shown are representative of 2 independent experiments. Click here for file

Additional file 3: Figure S3 Phenotype of HUT-102 cell line. Phenotype of HUT-102 cells were examined by FCM using anti-OX40 mAbs (FITC-labeled B-7B5 and Cy5-labeled W4-54), anti-OX40L (Cy5-labeled 5A8), biotinylated OX40 (rec-OX40) and OX40L (rec-OX40L) followed by PE-streptavidin. Intracellular Tax antigen was stained by mouse anti-Tax Lt-4 mAb. Click here for file
