# Supplementary figures and images for: Natural OX40L expressed on human T cell leukemia virus type-I-immortalized T cell lines interferes with infection of activated peripheral blood mononuclear cells by CCR5-utilizing human immunodeficiency virus
Source: Virol J. 2013 Nov 18;10:338. doi: 10.1186/1743-422X-10-338 (PMC4225675; doi:10.1186/1743-422X-10-338)

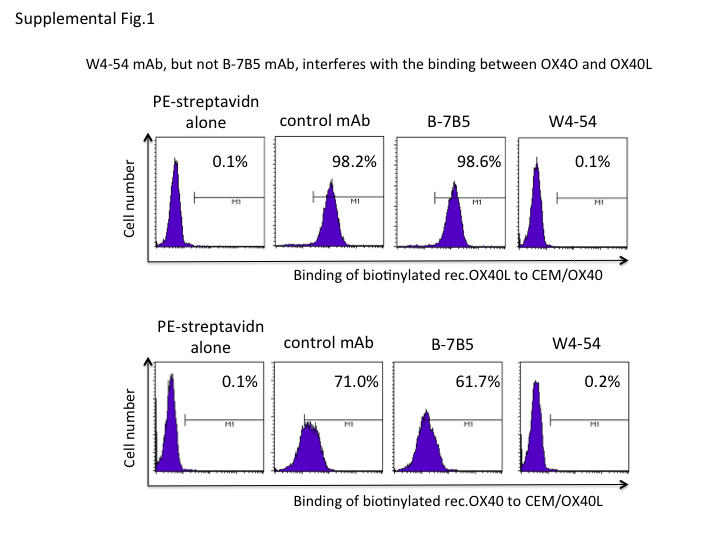

Supplement: Additional file 1: Figure S1 — Characterization of two anti-human OX40 mAbs. In the presence of B-7B5, W4-54 or isotype control mAbs, the OX40 and OX40L co-expressing control CEM cells were singly stained either with biotinylated recombinant OX40L (rec-OX40L) or rec-OX40, respectively, followed by PE-streptavidin. Data shown are representative profiles of 3 independent experiments. [file 1743-422X-10-338-S1.tiff]

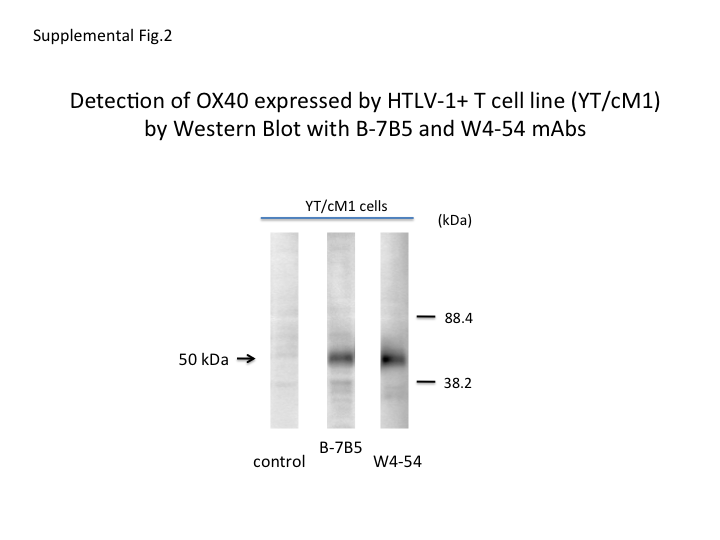

Supplement: Additional file 2: Figure S2 — Detection of OX40 expressed by HTLV-1+ T cell line (YT/cM1) by Western Blot with B-7B5 and W4-54 mAbs. Cell lysates of HTLV-1+ T cell line, YT/cM1, were subjected to 10% PAGE and blotted onto nitrocellulose sheets. The sheets were then probed with anti-OX40 mAbs (B-7B5 or W4-54) or isotype controls followed by goat anti-mouse IgG or anti-rat IgG. Mol. Wt. markers are shown on the right. Data shown are representative of 2 independent experiments. [file 1743-422X-10-338-S2.tiff]

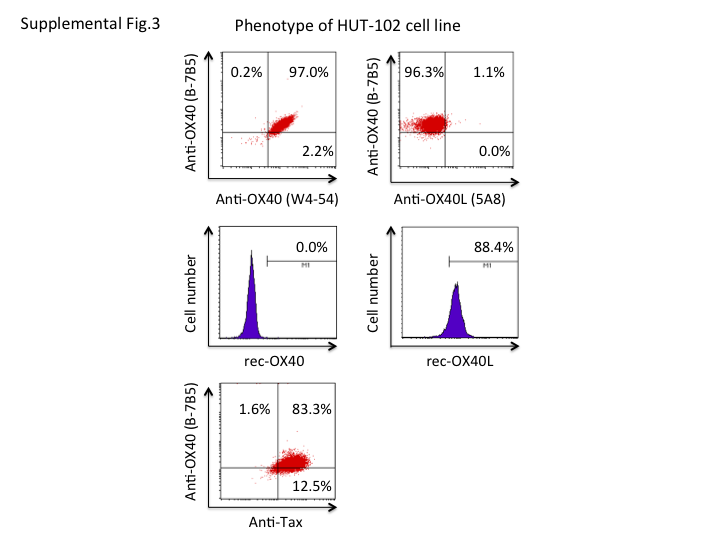

Supplement: Additional file 3: Figure S3 — Phenotype of HUT-102 cell line. Phenotype of HUT-102 cells were examined by FCM using anti-OX40 mAbs (FITC-labeled B-7B5 and Cy5-labeled W4-54), anti-OX40L (Cy5-labeled 5A8), biotinylated OX40 (rec-OX40) and OX40L (rec-OX40L) followed by PE-streptavidin. Intracellular Tax antigen was stained by mouse anti-Tax Lt-4 mAb. [file 1743-422X-10-338-S3.tiff]
